# The association of endometrial thickness with live birth: a differential impact of agonist vs antagonist ovarian stimulation protocols

**DOI:** 10.1530/RAF-25-0050

**Published:** 2026-09-02

**Authors:** Ruowu Ma, Xiaohui Ji, Xiangping Zhu, Meiqing Xie, Qingxue Zhang

**Affiliations:** ^1^ Center for Reproductive Medicine, Sun Yat-sen Memorial Hospital, Sun Yat-sen University, Guangzhou, China; ^2^ Center for Reproductive Medicine, Shenshan Medical Center, Sun Yat-sen Memorial Hospital, Sun Yat-sen University, Shanwei, China; ^3^ Guangdong Provincial Clinical Research Center for Obstetrical and Gynecological Diseases, Guangzhou, China

**Keywords:** downregulation, fresh embryo transfer, embryo quality, endometrial thickness, live birth

## Abstract

**Graphical Abstract:**

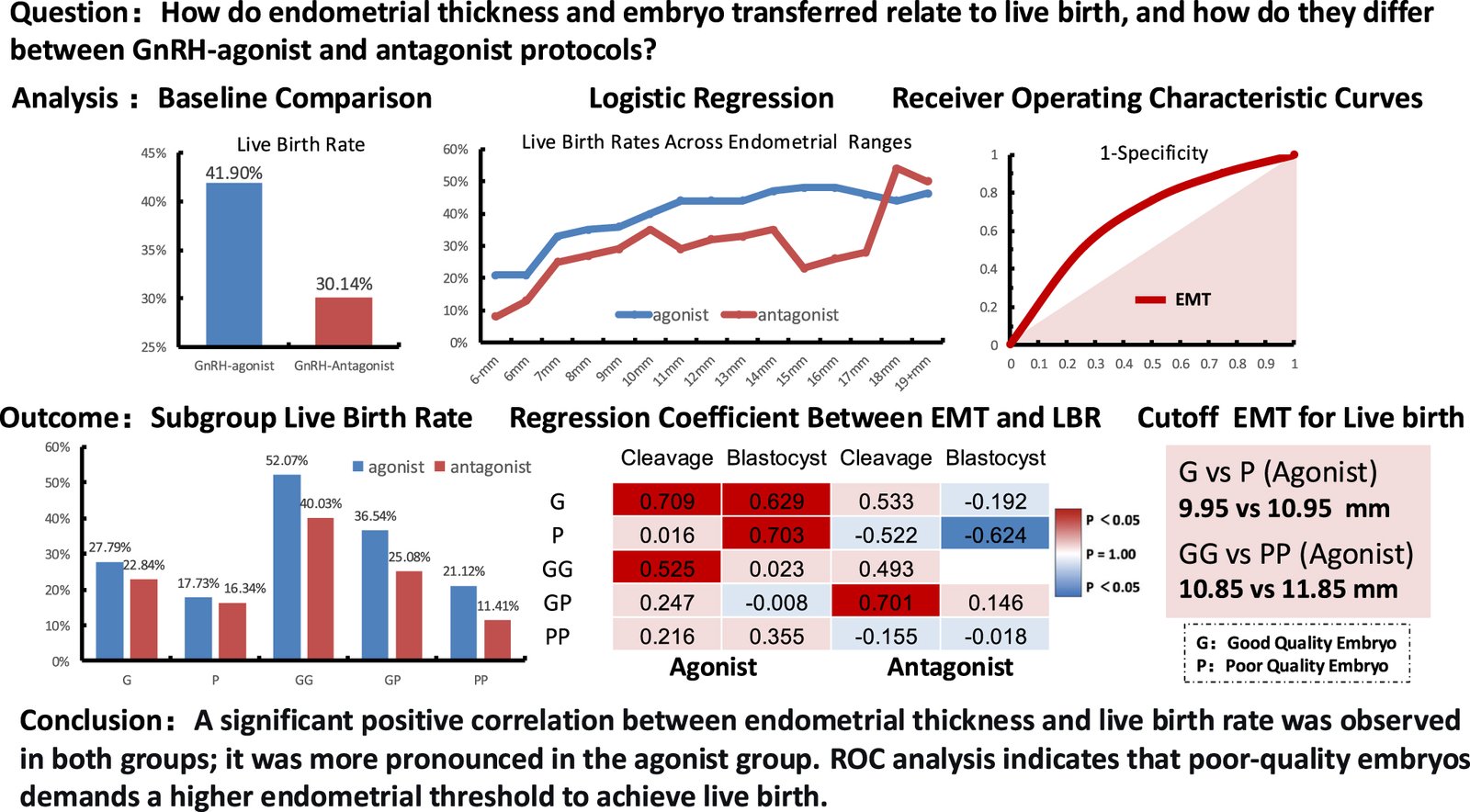

**Abstract:**

To explore the relationship between endometrium, quality of transferred embryo, and their effects on the live birth outcome of IVF/ICSI-ET using different downregulation protocols, we conducted a retrospective cohort study in an academic reproductive medical center. A total of 13,585 participant’s first fresh embryo transfer cycles, comprising 10,951 cycles using the GnRH-agonist regimen and 2,634 cycles using the GnRH-antagonist regimen, were included in this study. We found that the first fresh embryo transfer cycles using the GnRH-agonist downregulation regimen achieved a significantly improved live birth rate (41.9%) compared with the fresh cycles using the GnRH-antagonist regimen (30.4%). This was accompanied by a significantly thicker endometrium on the day of hCG administration. In the general population, increased endometrial thickness in the agonist group demonstrated a protective effect on the live birth. The cutoff value of endometrial thickness for live birth increased with the addition of poor-quality embryos in the double cleavage-stage embryo transfer cycles and the single blastocyst transfer cycles. Different choices of downregulation strategy affect the endometrium and the treatment outcome of IVF/ICSI-ET.

**Lay summary:**

The union of an embryo and the uterine lining is much like that of a seed and soil. The process of nurturing life resembles a seed taking root, sprouting, and bearing fruit. Different seeds require different conditions and soils. In assisted reproductive technology, the downregulation protocol used can affect the state of the uterine lining. Embryos of varying quality also have different chances of successful implantation. Just as fertile soil supports a seed’s growth, an endometrium of suitable thickness favors embryo implantation. When seed quality is less than ideal, a richer soil may improve its chance to take root. Similarly, when the available embryos are of lower quality, a thicker endometrial lining is associated with a higher live birth rate.

## Introduction

The embryo and endometrium are considered primary factors that may affect the prognosis of assisted reproductive technology (ART) treatment (Richter *et al.* 2007). Endometrial thickness (EMT) on the day of human chorionic gonadotropin (hCG) administration, number of oocytes retrieved, and embryo quality are all factors that need to be considered when determining the embryo transfer strategy (Huang *et al.* 2015). The study demonstrated the relationship between endometrial thickness and pregnancy rate following IVF/ICSI-ET (*in vitro* fertilization/intracytoplasmic sperm injection-embryo transfer). Researchers have declared that endometrial thickness may have predictive value for outcomes in the general population or certain specific subgroups (Zhang *et al.* 2005). The impact of downregulation choice on endometrial thickness and pregnancy was already noted in an earlier study (Kovacs *et al.* 2003). Therefore, we propose that the influence of the endometrial thickness on consequences of embryo transfer may follow a different pattern under different downregulation strategies using gonadotropin hormone-releasing hormone agonist (GnRH-agonist) or gonadotropin hormone-releasing hormone antagonist (GnRH-antagonist).

Since the embryo was another aspect of successful ART treatment, the transfer strategy with varying number and quality was continuously discussed (Hill *et al.* 2020). Herein, we explore the relationship between the number and quality of the transferred embryos, endometrial thickness on hCG day, and the live birth rate in the first fresh embryo transfer cycles using either GnRH-agonist or GnRH-antagonist as the downregulation regimen.

## Materials and methods

This was a retrospective study that reviewed the first IVF/ICSI-ET cycles at the Reproductive Medical Center of Sun Yat-sen Memorial Hospital from January 2011 to December 2020. This study was reviewed and approved by the Ethics Committee of Sun Yat-sen Memorial Hospital (SYSKY-2023-1020-01) on October 20, 2023, with retrospective registration. Informed consent was obtained from all individual participants included in the study.

The participants should meet the following criteria: undergoing IVF/ICSI-ET for the first time, use of the GnRH-agonist or GnRH-antagonist regimen, available embryos after oocyte retrieval, a fresh ET, and completion of birth follow-up. Cycles with mild stimulation and natural protocols, egg and sperm donation cycles, and cycles with no usable embryos or canceled fresh embryo transfers due to reasons such as the risk of ovarian hyperstimulation syndrome (OHSS) and cycles utilizing short-time GnRH-agonists as the trigger were not included. All participants received conventional and standard ART without any additional intervention.

For all cycles using the GnRH-agonist regimen, once downregulation was confirmed, controlled ovarian stimulation (COS) was initiated with a dose of 75–300 IU of recombinant gonadotrophin, depending on the participant’s age, body mass index (BMI), and ovarian reserve. In the antagonist group, ovarian stimulation was initiated on the second day of menstruation, and the GnRH-antagonist administration began on the fifth day of COS. For this, 4,000–10,000 IU of human chorionic gonadotrophin was injected when at least two follicles measured ≥18 mm. Transvaginal ultrasound-guided oocyte retrieval was performed 36 h after hCG administration, and fertilization was performed using either IVF or ICSI. The embryos were subsequently cultured until day 3 or 5.

Embryos at the cleavage stage or blastocyst stage that met certain criteria were defined as good-quality embryos (GQEs). The cleavage-stage embryo quality was evaluated according to the Cummins criteria (Cummins *et al.* 1986). The GQE should meet the following criteria: normal fertilization on the first day and normal morphology and size on the third day, with a total number of 6–12 blastomeres and a cellular debris ratio ≤20%. Blastocysts were graded per the Gardner system on day 5 of fertilization (Gardner *et al.* 2004), and the blastocysts that were superior to 4BB were defined as GQEs. The rest of the embryos that did not meet the GQE criteria were labeled as poor-quality embryos (PQEs). The number of embryo transfers was dependent on the patient age, ovarian reserve, number of usable embryos, and embryo quality.

Endometrial thickness was measured on hCG day using a transvaginal probe. The endometrial pattern was also recorded. Clinical pregnancy was confirmed by the visualization of the intrauterine gestational sac 4–6 weeks after ET. Live birth was defined as live neonate delivery after 28 weeks of gestation. Participants were categorized into the agonist group or antagonist group based on the downregulation protocol prescribed by their fertility specialist. Therefore, based on the number and quality of embryos transferred, each distinct group using different downregulation regimens was further divided into nine subgroups as follows: G: single-GQE transfer cycle; P: single-PQE transfer cycle; GG: double-GQE transfer cycle; GP: double-embryo transfer cycle, with one GQE and one PQE; PP: double-PQE transfer cycle; GGG: three-GQE transfer cycle; GGP: three-embryo transfer cycle, with two GQEs and one PQE; GPP: three-embryo transfer cycle, with one GQE and two PQEs; and PPP: three-PQE transfer cycle.

### Statistical analysis

The Kolmogorov–Smirnov test was performed to evaluate the normality of the data. Quantitative variables were analyzed either by Student’s *t*-test or by the Mann–Whitney *U* test depending on the distribution of the data. The chi-squared test was used to compare categorical variables, such as pregnancy rate and live birth rate. In some small-sized subgroups, we adopted the results of the Fisher exact test. A two-tailed *P* value of less than 0.05 was considered to be statistically significant. The baseline characteristics of participants among different groups were compared and included in the binary logistic regression using a forward stepwise likelihood ratio model, with live birth as the dependent variable. Before including these factors in the regression model, they all need to undergo a multicollinearity test. A linear regression model was used to assess the relationship between endometrial thickness and live birth rate. The regression coefficient (*β*) with its 95% confidence interval (CI) was reported to quantify the association. The coefficient of determination (*R*^2^) was calculated to evaluate the model fit. The cutoff values of the endometrial thickness affecting live birth were obtained using receiver operating characteristic (ROC) curve analysis. When there is heterogeneity of variance among multiple groups, we selected Dunn’s T3 for post hoc multiple comparisons. All statistical analyses were performed using SPSS, version 26 (IBM, USA).

## Results

This cohort included a total of 13,585 first fresh embryo transfer cycles, comprising 10,951 cycles using the GnRH-agonist regimen and 2,634 cycles using the GnRH-antagonist regimen. The age of participants ranged from 20 to 51 years. The number of transferred embryos ranged from 1 to 3. In the agonist group, the general clinical pregnancy rate and live birth rate were 59.32% (6,496/10,951) and 41.90% (4,588/10,951), respectively. In the antagonist group, the clinical pregnancy rate and live birth rate were 50.30% (1,325/2,634) and 30.14% (794/2,634), respectively. The baseline characteristics of participants, such as age and years of infertility, and the relevant outcomes, such as number of oocytes retrieved, exhibited significant differences between these two groups (Table 1). As a result, we performed a separate analysis according to the cycle’s downregulation protocol.

**Table 1:** Comparison of general characteristics between the agonist and antagonist groups. Values are presented as mean ± SD.

	Agonist group	Antagonist group	*P* value
Female age (years)	32.50 ± 4.53	32.34 ± 5.31	<0.001
Years of infertility	4.47 ± 3.25	4.28 ± 3.12	<0.001
Baseline FSH (IU/L)	8.13 ± 2.93	8.02 ± 3.36	<0.001
Baseline LH (IU/L)	4.92 ± 3.52	6.17 ± 4.77	<0.001
Baseline AMH (ng/mL)	3.94 ± 2.90	5.8 ± 4.85	<0.001
Gonadotropin dose in COS (IU)	2,355.76 ± 837.92	1750.59 ± 745.37	<0.001
COS duration (day)	11.88 ± 3.85	9.68 ± 2.66	0.663
FSH on hCG day (IU/L)	18.98 ± 7.72	16.43 ± 7.97	0.701
LH on hCG day (IU/L)	1.84 ± 1.60	3.55 ± 3.84	<0.001
P on hCG day (ng/mL)	1.09 ± 0.85	1.11 ± 1.60	0.573
EMT on hCG day (mm)	11.79 ± 2.73	10.86 ± 2.35	<0.001
Oocytes retrieved	11.13 ± 5.23	10.62 ± 5.38	0.001
Embryo transferred	1.89 ± 0.51	1.74 ± 0.52	<0.001
Pregnancy rate	59.32%	50.30%	<0.001
Live birth rate	41.90%	30.14%	<0.001
Multiple live birth rate	12.28%	6.61%	<0.001

FSH, follicular-stimulating hormone; LH, luteinizing hormone; AMH, anti-Müllerian hormone; COS, controlled ovarian stimulation; hCG, human chorionic gonadotrophin; P, progesterone; EMT, endometrial thickness.

Within each of the agonist and antagonist groups, we compared the variables in Table 1 between cycles with and without a live birth. Significant differences also remained (Table S1 (see section on Supplementary materials given at the end of the article)). Then, we constructed a binary logistic regression model, with live birth as the outcome using the following variables: female age, infertility years, baseline FSH, baseline LH, baseline AMH, total gonadotropin (Gn) dose, FSH on hCG day, LH on hCG day, endometrial thickness on hCG day, number of oocytes retrieved, and number of embryos transferred. The variables retained in the final model are presented in Table 2, while those excluded from the regression analysis are listed in Supplementary Table 3. In the GnRH-agonist group, an increased thicker endometrial thickness on hCG day was found to have a protective effect on live birth. In both groups, the age and number of embryos transferred were included in the regression model (Table 2).

**Table 2:** Binary logistic regression analysis with live birth as the dependent variable.

Variable	Agonist group				Antagonist group			
	*β*	OR	95% CI	*P*	*β*	OR	95% CI	*P*
Female age	−0.092	0.918	0.906–0.930	<0.001	−0.066	0.936	0.914–0.958	<0.001
Years of infertility	−0.017	0.985	0.966–1.004	0.057	−0.063	0.939	0.905–0.973	0.001
EMT on hCG day	0.045	1.046	1.026–1.066	<0.001	−0.002	0.998	0.989–1.007	0.618
Embryo transferred	0.799	2.088	1.785–2.303	<0.001	0.624	1.867	1.517–2.217	<0.001

*β*, standard regression coefficient; OR, odds ratio; CI, confidence interval; EMT, endometrial thickness.

**Figure 1:**
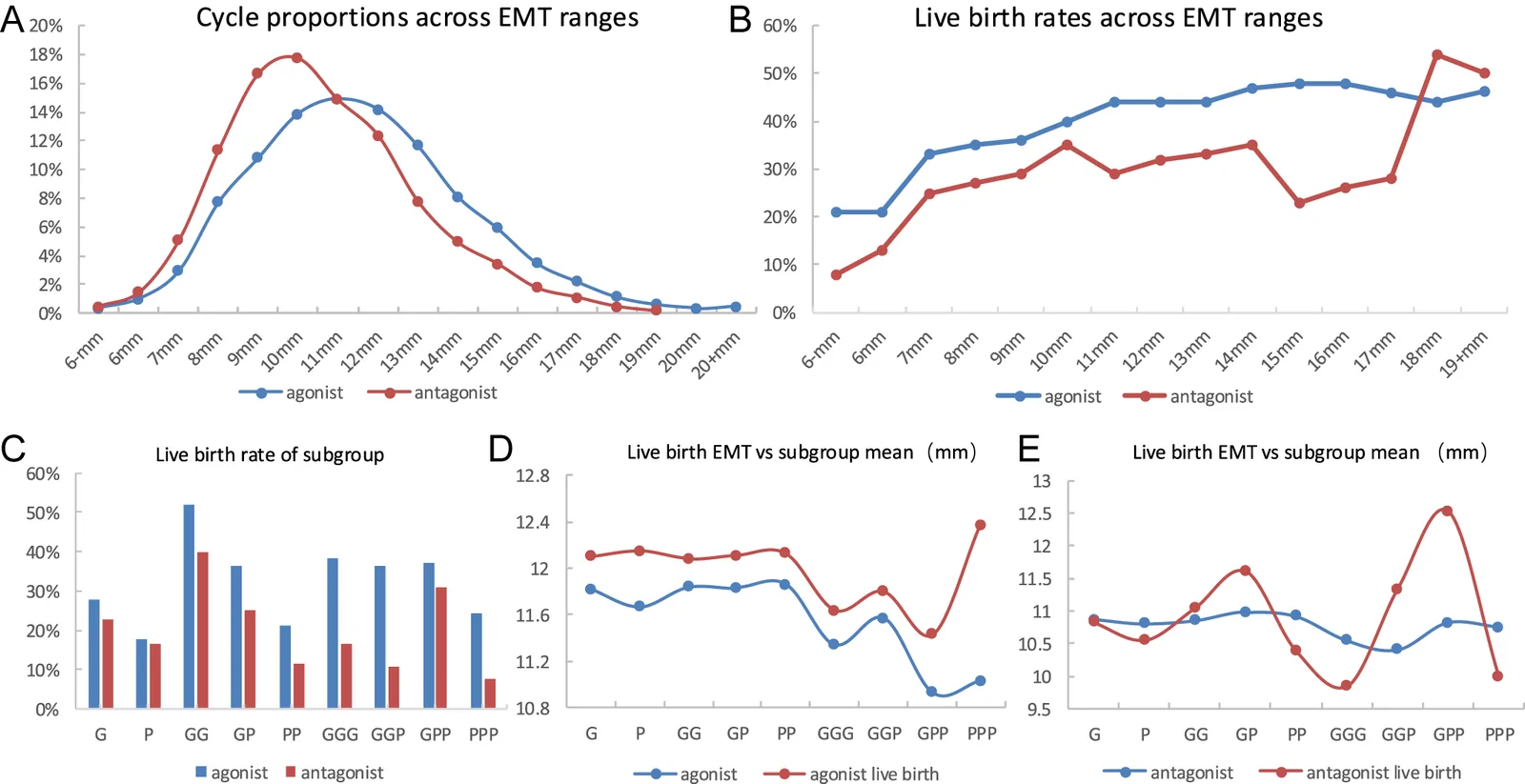
General description of the differences between the GnRH-agonist group and the GnRH-antagonist group. (A) The endometrial thickness on hCG day followed a normal distribution in both groups. (B) A significant positive correlation was observed between endometrial thickness and live birth rate both in the agonist group (*β* = 0.858; *R*^2^ = 0.736; *P* < 0.001) and in the antagonist group (*β* = 0.733; *R*^2^ = 0.537; *P* = 0.002). (C) Comparison of live birth rates between protocols within embryo quality subgroups. (D and E) Comparison of endometrial thicknesses between live birth cycles and the overall average within the (D) agonist and (E) antagonist groups.

In both the agonist and antagonist groups, the endometrial thickness on hCG day followed a normal distribution (Fig. 1A) and it was significantly higher in the agonist group (11.79 ± 2.73 mm) compared with the antagonist group (10.86 ± 2.35 mm) (Table 1). In the agonist group, the lowest endometrial thickness resulting in pregnancy and live birth was 2.7 mm in a patient who received a single good-quality cleavage-stage embryo. In the antagonist group, it was 5.9 mm and, similarly, from a single good-quality cleavage-stage embryo transfer cycle.

Across the entire endometrial thickness range, it was evident that the live birth rate gradually increased with a thicker endometrium (Fig. 1B, and a significant positive correlation between endometrial thickness and live birth rate was observed in both groups; it was more pronounced in the agonist group (*β* = 0.858, *R*^2^ = 0.736, *P* < 0.001) than in the antagonist group (*β* = 0.733, *R*^2^ = 0.537, *P* = 0.002). In the subgroup sharing the same embryo quality and quantity, the live birth rate in the agonist group was higher than in the antagonist group. This difference was statistically significant in six of the nine subgroups, excluding P, GPP, and PPP (Fig. 1C) (Table S2). When comparing the endometrial thickness resulting in live birth among different subgroups, it was observed that in the agonist group, the endometrial thickness of live birth cycles was higher than the subgroup’s overall average (Fig. 1D). However, in the antagonist group, this pattern appeared to be less pronounced (Fig. 1E).

### GnRH-agonist group

**Figure 2:**
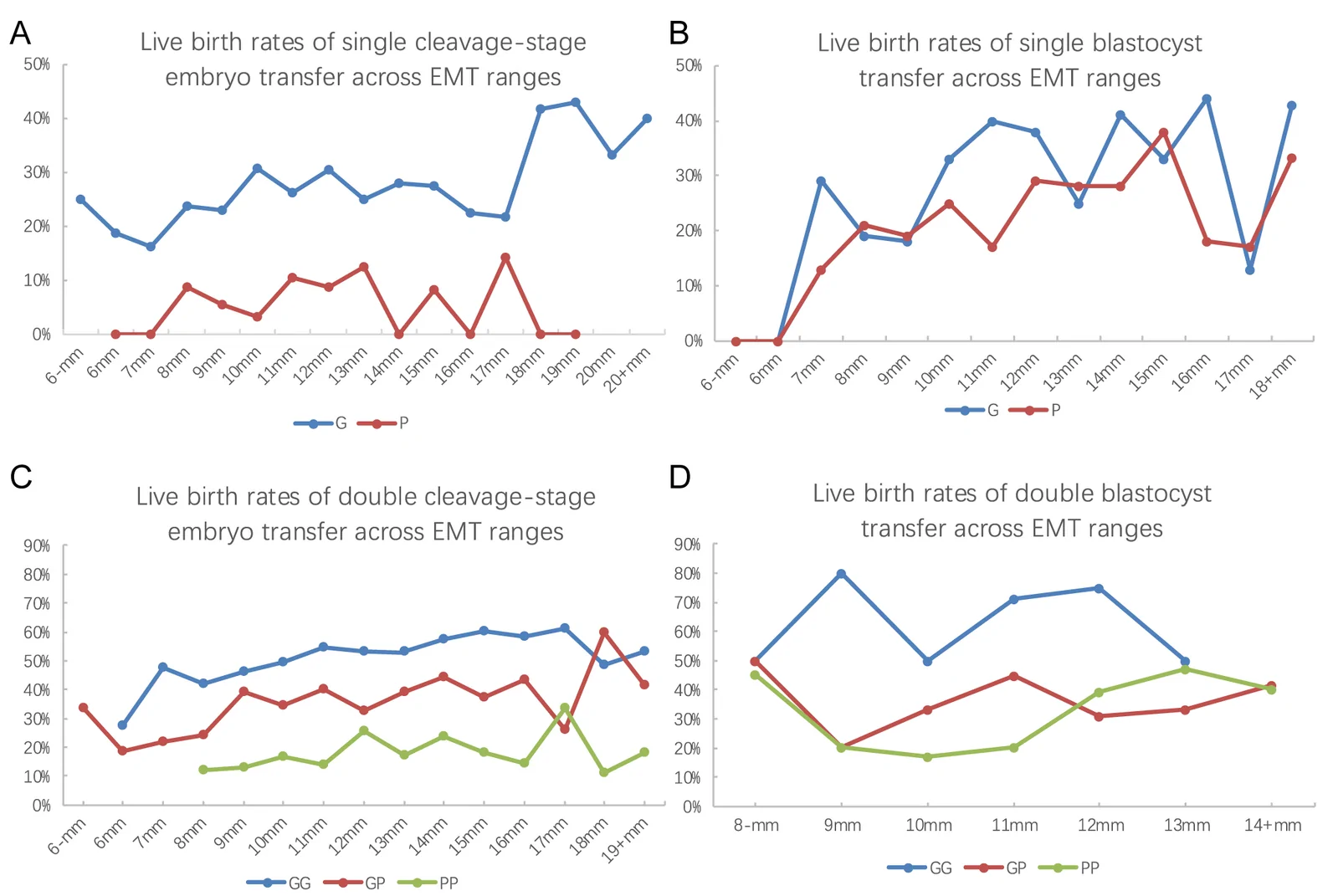
Association between endometrial thickness and live birth rate in the GnRH-agonist group, stratified by embryo type, quality, and number transferred. (A) Single cleavage-stage embryo: a significant positive correlation was observed with good-quality embryos (G: *β* = 0.709, *R*^2^ = 0.503, *P* = 0.001), but not with poor-quality embryos (P: *β* = 0.016, *R*^2^ < 0.001, *P* = 0.957). (B) Single blastocyst: a significant positive correlation was observed for both good-quality (G: *β* = 0.629, *R*^2^ = 0.395, *P* = 0.016) and poor-quality (P: *β* = 0.703, *R*^2^ = 0.495, *P* = 0.005) embryos. (C) Double cleavage-stage embryos: a significant correlation was observed only with double good-quality embryo transfer (GG: *β* = 0.525, *R*^2^ = 0.276, *P* = 0.044), not with mixed (GP: *β* = 0.247, *P* = 0.340) or double poor-quality (PP: *β* = 0.216, *P* = 0.458) embryos. (D) Double blastocysts: no significant association was found in any group (GG: *β* = 0.023, *P* = 0.966; GP: *β* = −0.008, *P* = 0.987; PP: *β* = 0.355, *P* = 0.434).

In the single cleavage-stage embryo transfer cycles, we observed significant differences in live birth rates between the subgroup G and the subgroup P (*P* < 0.001), and the live birth rate of the GQE transfer cycles was consistently higher than that of PQE transfer cycles in their overall endometrial thickness range (Fig. 2A). A significant positive correlation was detected between endometrial thickness and live birth rate in the subgroup G (*β* = 0.709, *R*^2^ = 0.503, *P* = 0.001) but not in the subgroup P. When transferred with two cleavage-stage embryos, the subgroups also exhibited significant differences in live birth rates (*P* < 0.001). A significant correlation between endometrial thickness and live birth rate was only detected in the subgroup GG (*β* = 0.525, *R*^2^ = 0.276, *P* = 0.044), and GG exhibited a consistently higher live birth rate than PP, with the GP group falling between GG and PP (Fig. 2C).

In the blastocyst transfer cycles, significant differences between the live birth rates remained when comparing the single-blastocyst transfer cycle subgroups G and P (*P* = 0.019) or the double-blastocyst subgroups GG, GP, and PP (*P* = 0.010). A significant positive correlation between endometrial thickness and live birth rate was detected in the single-blastocyst transfer cycle subgroups G (*β* = 0.629, *R*^2^ = 0.395, *P* = 0.016) and P (*β* = 0.703, *R*^2^ = 0.495, *P* = 0.005), but not in the double-blastocyst transfer cycles (Fig. 2B and D). Single poor-quality blastocyst transfer cycles showed significantly higher pregnancy and live birth rates compared with single poor-quality cleavage-stage embryo transfer cycles (*P* < 0.001). When the thickness of the endometrium was less than 7 mm, the subgroups in which solely poor-quality embryos were transferred (P, PP, and PPP) using the GnRH-agonist regimen did not result in any live births.

When more poor-quality cleavage-stage embryos were included, the subgroup PP might achieve a better prognosis than the subgroup P because the pregnancy rate increased significantly from 14.88 to 31.97% (*P* < 0.001) and the live birth rate increased significantly from 6.98 to 17.35% (*P* = 0.010) (Table 3), across the entire endometrial thickness range (Fig. S1B). Compared with the subgroup G, there was a significant increase in the pregnancy rate and live birth rate in the subgroup GG (*P* < 0.001). However, it also contributed to a significant increase in the multiple birth rate (*P* < 0.001). It is important to highlight that both the pregnancy rate and the live birth rate experienced a significant decrease in the subgroup GGG compared with the GG subgroup (Table 3) (Table S4). Additionally, in the majority of the endometrial thickness range, the live birth rates of GGG fell between GG and G (Fig. S1A).

**Table 3:** Number of cycles, clinical pregnancy rates, and multiple birth rates in different quality subgroups of cleavage-stage embryos and blastocysts within the agonist group.

Subgroup	Cleavage-stage embryo				Blastocyst			
	Cycles	CPR	LBR	MBR	Cycles	CPR	LBR	MBR
G	1,110	46.49%	26.67%	0.27%	376	68.35%	30.59%	1.33%
P	215	14.88%	6.98%	0.00%	394	47.46%	23.60%	0.00%
GG	5,999	68.38%	52.01%	18.20%	26	80.77%	65.38%	34.62%
GP	1,249	53.48%	36.51%	7.93%	62	58.06%	37.10%	17.74%
PP	513	31.97%	17.35%	3.70%	150	52.67%	34.00%	9.33%
GGG	405	53.33%	38.27%	13.58%	0			
GGP	261	52.11%	36.02%	9.58%	1	100.00%	100.00%	0.00%
GPP	115	48.70%	37.39%	11.30%	1	0.00%	0.00%	0.00%
PPP	68	29.41%	19.12%	4.41%	6	83.33%	83.33%	16.67%

CPR, clinical pregnancy rate; LBR, live birth rate; MBR, multiple birth rate.

In the blastocyst transfer cycles, the GG subgroup achieved a significantly higher live birth rate (65.38%) compared with the G subgroup (30.59%) (Table 3) (Table S4). However, it was also associated with the highest multiple birth rate (34.62%). The inclusion of poor-quality blastocyst embryos in the PP subgroup resulted in increased clinical pregnancy rates and live birth rates compared with the subgroup P, but the differences were not significant.

We obtained the cutoff values of the endometrial thickness affecting live birth through ROC curve analysis. Although all subgroups had an area under the curve (AUC) of greater than 0.5 (Table 4), we applied a more stringent control based on the lower limit of the 95% confidence interval (CI) and found that only in the cleavage-stage embryo transfer subgroups GG, GP, and PP, as well as in the blastocyst subgroups G and P, the cutoff values of the endometrial thickness on hCG day had discriminative significance for live birth outcomes. In the PP subgroup transferred with two poor-quality cleavage-stage embryos, the cutoff value was 11.85 mm, and in the GG subgroup, it was 10.85 mm. In the blastocyst transfer cycles, the cutoff value for the subgroup P was 11.85 mm, and for the subgroup G, it was only 9.65 mm. In certain embryo transfer cycles, such as double cleavage-stage embryo or single-blastocyst transfer cycles, the ROC curve analysis indicated that the increased endometrial thickness is associated with an improved live birth rate when poor-quality embryos were transferred.

**Table 4:** Correlation analysis between EMT and live birth on the ROC curve in different subgroups of embryo transfer cycles using GnRH-agonist regimen. The three-blastocyst transfer cycles were not plotted due to their small sample size.

Subgroup	Cleavage-stage embryo				Blastocyst			
	AUC	*P* value	95% CI	Cutoff (mm)	AUC	*P* value	95% CI	Cutoff (mm)
G	0.525	0.203	0.487–0.563	9.95	0.567	0.038	0.506–0.627	9.65
P	0.53	0.072	0.388–0.671	10.95	0.567	0.049	0.501–0.634	11.85
GG	0.555	0.000	0.540–0.570	10.85	0.526	0.829	0.274–0.778	9.25
GP	0.546	0.006	0.514–0.579	10.35	0.556	0.648	0.419–0.627	9.05
PP	0.571	0.036	0.509–0.633	11.85	0.556	0.462	0.407–0.706	11.45
GGG	0.552	0.076	0.495–0.610	10.05				
GGP	0.649	0.203	0.481–0.837	12.85				
GPP	0.547	0.115	0.476–0.619	9.90				
PPP	0.588	0.097	0.480–0.696	12.75				

ROC, receiver operating characteristic; AUC, area under the curve; CI, confidence interval.

### GnRH-antagonist group

**Figure 3:**
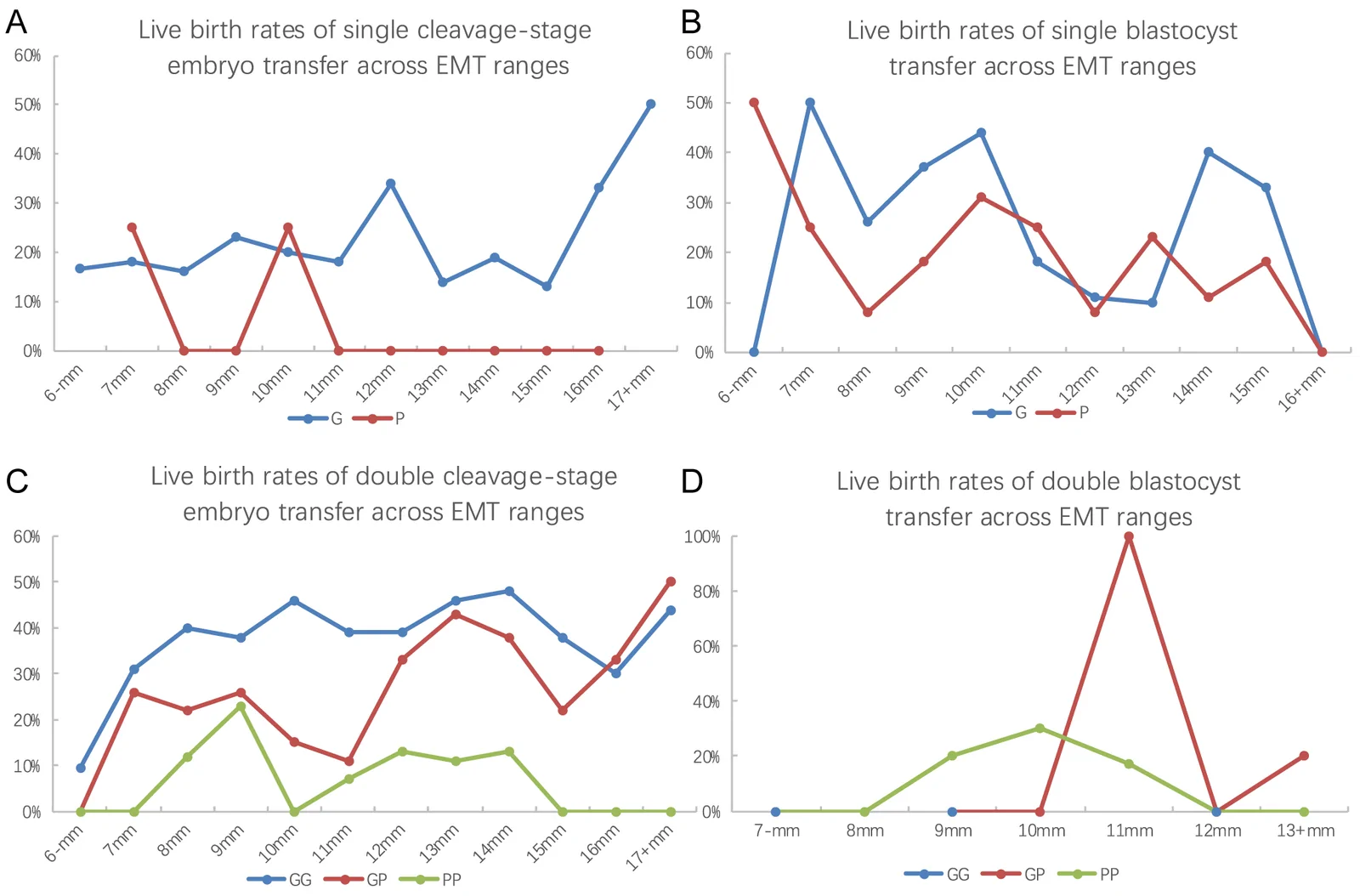
Association between endometrial thickness and live birth rate in the GnRH-antagonist group, stratified by embryo type, quality, and number transferred. (A and C) Cleavage-stage embryos: a significant linear correlation was only observed in the group of mixed embryo quality (GP: *β* = 0.701, *R*^2^ = 0.492, *P* = 0.011); (B and D) Blastocysts: a significant negative correlation was observed in the single poor-quality embryo group (P: *β* = −0.624, *R*^2^ = 0.389, *P* = 0.040).

In the GnRH-antagonist group, the relationship between endometrial thickness and live birth rate seemed less pronounced in single-embryo transfer cycles. A significant correlation was only detected in the single poor-quality blastocyst transfer cycles (*β* = −0.624, *R*^2^ = 0.389, *P* = 0.040) (Fig. 3B; Table 5). Within the antagonist group, only three cycles involved the transfer of two good-quality embryos at the blastocyst stage. These three cycles had endometrial thicknesses of 6, 9, and 12 mm, respectively. Among them, one cycle resulted in a biochemical pregnancy, and the other two cycles experienced early spontaneous miscarriages. Consequently, the curve for the blastocyst GG group is not visible in Fig. 3D.

**Table 5:** Number of cycles, pregnancy rates, and multiple birth rates in different quality subgroups of cleavage-stage embryos and blastocysts in the antagonist group. No three-blastocyst transfer cycle was included in the antagonist group.

Subgroup	Cleavage-stage embryo				Blastocyst			
	Cycles	CPR	LBR	MBR	Cycles	CPR	LBR	MBR
G	421	39.43%	21.38%	0.00%	157	57.96%	26.75%	0.00%
P	59	15.25%	6.78%	0.00%	143	43.36%	20.28%	0.00%
GG	1,291	61.04%	40.12%	12.08%	3	66.67%	0.00%	0.00%
GP	300	41.00%	24.67%	3.33%	15	53.33%	33.33%	13.33%
PP	118	26.27%	10.17%	1.69%	31	35.48%	16.13%	3.23%
GGG	42	30.95%	16.67%	0.00%				
GGP	28	32.14%	10.71%	3.57%				
GPP	13	46.15%	30.77%	15.38%				
PPP	13	38.46%	7.69%	0.00%				

CPR, clinical pregnancy rate; LBR, live birth rate; MBR, multiple birth rate.

Our findings indicated that within the subgroup of cleavage-stage embryo transfer cycles in the antagonist group, GG was associated with a significantly higher pregnancy rate (61.04%), live birth rate (40.12%), and multiple birth rate (12.08%) compared with the subgroup G (39.43, 21.38, and 0%, respectively) (*P* < 0.001). However, the pregnancy rate (30.95%) and live birth rate (16.67%) in the subgroup GGG were significantly lower than those in the GG subgroup (*P* = 0.003, *P* = 0.01)(Table 5)(Table S5). This was similar to the change in the agonist group with cleavage-stage embryo transfer. Unlike the agonist group, the addition of PQEs contributed to a live birth rate reduction in the double poor-quality blastocyst embryo transfer cycles (16.13%) compared with the single poor-quality blastocyst transfer cycles (20.28%). In the corresponding cleavage-stage embryo transfer cycles, PP showed an insignificant increase in the live birth rate (10.17%) compared with P (6.78%). We also observed a reduction in the live birth rate in the three poor-quality cleavage-stage embryo transfer cycles (7.69%) compared with the double-PQE transfer cycles. In the antagonist group, the addition of PQEs at the cleavage stage in the subgroup GP did not result in a significantly improved live birth rate compared with the subgroup G (Table S5).

When plotting the ROC curves, we noticed that endometrial thickness on hCG day had predictive value for live birth (AUC > 0.5) only in a few subgroups (Table S6). For the cleavage-stage embryo transfer cycles within the antagonist group, the cutoff values for endometrial thickness in the GG and GP subgroups were 9.05 mm (AUC: 0.535, 95% CI: 0.505–0.568) and 11.95 mm (AUC: 0.584, 95% CI: 0.504–0.664), respectively.

## Discussion

Pituitary desensitization to prevent premature luteinization is crucial for the success of COS. Both the GnRH-agonist and GnRH-antagonist protocols are current mainstream choices in the general IVF population. The GnRH-agonist protocol was reported to improve the pregnancy rate (Lambalk *et al.* 2017) and live birth rate (Yang *et al.* 2021) compared with the GnRH-antagonist protocol in the general IVF population due to its endometrial receptivity improvement (Geng *et al.* 2019). The GnRH-antagonist protocol was reported to contribute a lower incidence of OHSS (Al-Inany *et al.* 2016).

In this cohort, we found that the participants in the agonist group, despite having less favorable baseline characteristics, achieved a significantly higher pregnancy rate and live birth rate compared with those in the antagonist group. Within the subgroups sharing the same quantity and quality embryos, the agonist group achieved a higher live birth rate in all subgroups, with significance in six of the nine subgroups. These advantages remained consistent in both cleavage-stage embryo and blastocyst transfer cycles within each subgroup. This difference may be attributed to the effect of the downregulation regimen on endometrial receptivity. Earlier studies have reported that GnRH-antagonists impact endometrial stromal and epithelial cells, which may impair endometrial receptivity (Chen *et al.* 2020, Xu *et al.* 2022). However, this impairment might be eliminated by changing the transfer strategy (Liu *et al.* 2019). Frozen–thawed embryo transfer using the GnRH-antagonist protocol may achieve a 1.48 times higher clinical pregnancy rate than a fresh transfer cycle, with an improvement in subendometrial blood flow (Yu *et al.* 2023).

The effect of GnRH-agonists and clomiphene citrate on endometrium and pregnancy outcome was discussed in the study by Kovacs *et al.* (2003). Since downregulation may affect endometrial receptivity, and a significant difference in endometrial thickness was observed in our study between the agonist and antagonist groups, we analyzed and discussed the relationship between endometrial thickness and live birth in these two groups separately. It has been reported that increased endometrial thickness can improve clinical pregnancy outcomes in normal responders using GnRH-agonists or GnRH-antagonists (Wu *et al.* 2014, Luo *et al.* 2021). Figure 1B clearly shows that live birth rates gradually increased with endometrial thickness in the general population of the agonist group, while in the antagonist group, within the range of 13–17 mm, there was a decrease in the live birth rate.

When constructing the binary logistic regression model with live birth as the dependent variable, endometrial thickness was not included in the regression model for the antagonist group, nor did it show significance between the live birth cycles and non-live birth cycles (*P* = 0.116) (Table S1). The correlation between endometrial thickness and live birth rate was also less pronounced in the antagonist group (Fig. 1B).

Subsequently, we examined participants aged 40 years and above, and endometrial thickness showed a significant negative effect on live birth outcome (*β* = −0.348, OR: 0.706, 95% CI: 0.539–0.925, *P* = 0.011), but not in the agonist group. When the live birth rate changing across the endometrial thickness range was plotted, the live birth rates of the antagonist group decreased gradually when the endometrial thickness was greater than 11 mm (Fig. S1). Meanwhile, the general mean of antagonist live birth cycles was 11.02 ± 2.27 mm (Table S1). Zhang’s study reported that the optimal endometrial thickness was 8.7–14.5 mm in HRT-FET cycles and live birth rates may decrease when the endometrium is either too thick or too thin (Shaodi *et al.* 2020). From the predictive model and the decreasing live birth rates in certain endometrial thickness range, we believed that for participants older than 40 years using GnRH-antagonists, an endometrial thickness of greater than 11 mm might indicate an unfavorable outcome.

Insufficient endometrial thickness on hCG day may serve as an indicator of unfavorable pregnancy outcomes. A thickness of 7 mm has frequently been reported as a cutoff value associated with a lower chance of achieving pregnancy (Kasius *et al.* 2014). An endometrial thickness of ≤7.5 mm has been considered to be associated with an increased risk of poor placentation-related obstetric complications (Oron *et al.* 2018), and the risk of having small-for-gestational-age (SGA) infants doubles compared with an endometrial thickness >12 mm (Guo *et al.* 2020).

In this cohort, there were a total of 351 cycles with an endometrial thickness of ≤7 mm on hCG day. The live birth rate in the agonist group (25.37%, 69/272) was higher than that in the antagonist group (16.46%, 13/79) without statistical significance (*P* = 0.099). In thin-endometrium transfer cycles, the GG group ranked first in both the total cycle number and live birth rate between the two groups, with rates of 35.77% (44/123) in the agonist group and 16.67% (8/48) in the antagonist group. However, when transferring a single poor-quality embryo in the thin-endometrium range, the live birth rate in the agonist group (6.25%, 1/16) was lower than that in the antagonist group (25%, 1/4).

From past studies, the addition of PQEs has been discussed, and in certain studies, it was not recommended when a GQE was available in both cleavage-stage embryo and blastocyst transfer cycles (Zhu *et al.* 2020). We performed a ROC curve analysis for endometrial thickness on hCG day in relation to live birth outcome for each subgroup and discussed cleavage-stage embryo and blastocyst separately. In the cycles of the agonist group involving embryo transfers at the cleavage stage, the inclusion of PQEs was associated with a significant increase in pregnancy rates and live birth rates (G vs GP; P vs PP). However, in the antagonist group, the increase in pregnancy rates and live birth rates was mild and not significant. In the blastocyst transfer cycles of the antagonist group, the addition of PQEs (PP vs P) resulted in a decrease in pregnancy rates and live birth rates. In both the antagonist and agonist groups, it is not recommended to transfer three good-quality cleavage-stage embryos because in both groups, the pregnancy rate and live birth rate for GGG were significantly lower than those for GG. Considering the results of both binary logistic regression and ROC curve analyses, we concluded that generally, the endometrial thickness on hCG day has more predictive value for live birth outcome in the first fresh embryo transfer cycle using an agonist regimen. Additionally, a thicker endometrium was required to achieve live birth in certain subgroups using agonists or antagonists.

## Conclusion

Under the condition of transplanting the same quantity and quality of embryos, the fresh embryo transfer cycles using the GnRH-agonist regimen yielded a better live birth outcome. The endometrial thickness affects the live birth outcome in a different pattern in the fresh cycles using different downregulation regimens; the association was most pronounced in GnRH-agonist cycles with fresh good-quality embryo transfer. In the antagonist group, the inclusion of poor-quality blastocysts in the fresh transfer cycles contributed to less favorable effects on live birth outcome compared with the agonist group. An increasing embryo transfer number or a decreasing embryo quality required a greater endometrial thickness to achieve live birth.

## Limitations

This is a retrospective study, and selection bias and unmeasured confounding factors cannot be completely ruled out. Given that the GnRH-agonist and GnRH-antagonist protocols are the most widely used regimens, we selected fresh cycles from these two protocols for analysis. Cycles using mild stimulation, natural cycle, luteal-phase stimulation, or other protocols were not included. Because the agonist and antagonist groups were not directly comparable due to inherent differences in baseline characteristics, we performed the analysis and discussion separately within each group. We did not present the estradiol concentration, as the assay used for estradiol had an upper detection limit of 4,800 ng/L. In about 12% of all the cycles, peak E_2_ levels exceeded this value and were recorded as semiquantitative (>4,800 ng/L). The sample size of three-embryo transfer cycles was rather limited, and when we separately discussed the conditions of the cleavage-stage embryo and blastocyst transfer cycles, certain subgroups were missing. Obstetric complications were not discussed in this study.

## Supplementary materials





## Declaration of interest

The authors declare that there is no conflict of interest that could be perceived as prejudicing the impartiality of the research reported.

## Funding

This study was supported by the Sun Yat-sen University Clinical Research 5010 Program (Grant No. 2016004) and the National Natural Science Foundation of China (Grant Nos. 82171642, 81971332, and 82171670).

## Author contribution statement

RWM collected and analyzed the data and drafted the manuscript. XHJ and XPZ helped to analyze the data and performed a technical check. MQX and QXZ designed the study and revised the manuscript. All authors critically revised the manuscript, contributed to the submission, and approved the version to be published. All authors agreed on the presented findings and the contents of the final draft and take responsibility for the authenticity of the data used in the paper.

## Data availability

All original data involved in this research have been uploaded and surveyed in REDCap (https://redcap.gzsys.org.cn:8082/) and RDD (http://rdd.sysu.edu.cn). The data are accessible solely upon a reasonable request.

## References

[bib1] Al-Inany HG, Youssef MA, Ayeleke RO, et al. 2016 Gonadotrophin-releasing hormone antagonists for assisted reprod technology. Cochrane Database Syst Rev 2016 CD001750. (10.1002/14651858.cd001750.pub4) PMC8626739 27126581

[bib2] Chen Q, Fan Y, Zhou X, et al. 2020 GnRH antagonist alters the migration of endometrial epithelial cells by reducing CKB. Reproduction 159 733–743. (10.1530/rep-19-0578) 32213653

[bib3] Cummins JM, Breen TM, Harrison KL, et al. 1986 A formula for scoring human embryo growth rates in in vitro fertilization: its value in predicting pregnancy and in comparison with visual estimates of embryo quality. J In Vitro Fert Embryo Transf 3 284–295. (10.1007/bf01133388) 3783014

[bib4] Gardner DK, Surrey E, Minjarez D, et al. 2004 Single blastocyst transfer: a prospective randomized trial. Fertil Steril 81 551–555. (10.1016/j.fertnstert.2003.07.023) 15037401

[bib5] Geng Y, Xun Y, Hu S, et al. 2019 GnRH antagonist versus follicular-phase single-dose GnRH agonist protocol in patients of normal ovarian responses during controlled ovarian stimulation. Gynecol Endocrinol 35 309–313. (10.1080/09513590.2018.1528221) 30430883

[bib6] Guo Z, Xu X, Zhang L, et al. 2020 Endometrial thickness is associated with incidence of small-for-gestational-age infants in fresh in vitro fertilization-intracytoplasmic sperm injection and embryo transfer cycles. Fertil Steril 113 745–752. (10.1016/j.fertnstert.2019.12.014) 32147172

[bib7] Hill MJ, Eubanks AE, Csokmay JM, et al. 2020 Is transferring a lower-quality embryo with a good- quality blastocyst detrimental to the likelihood of live birth? Fertil Steril 114 338–345. (10.1016/j.fertnstert.2020.03.027) 32624214

[bib8] Huang Y, Wang EY, Du QY, et al. 2015 Progesterone elevation on the day of human chorionic gonadotropin administration is not the only factor determining outcomes of in vitro fertilization. Fertil Steril 103 106–111. (10.1016/j.fertnstert.2014.10.019) 25455869

[bib9] Kasius A, Smit JG, Torrance HL, et al. 2014 Endometrial thickness and pregnancy rates after IVF: a systematic review and meta-analysis. Hum Reprod Update 20 530–541. (10.1093/humupd/dmu011) 24664156

[bib10] Kovacs P, Matyas S, Boda K, et al. 2003 The effect of endometrial thickness on IVF/ICSI outcome. Hum Reprod 18 2337–2341. (10.1093/humrep/deg461) 14585884

[bib11] Lambalk CB, Banga FR, Huirne JA, et al. 2017 GnRH antagonist versus long agonist protocols in IVF: a systematic review and meta-analysis accounting for patient type. Hum Reprod Update 23 560–579. (10.1093/humupd/dmx017) 28903472

[bib12] Liu X, Bai H, Shi W, et al. 2019 Frozen-thawed embryo transfer is better than fresh embryo transfer in GnRH antagonist cycle in women with 3-10 oocytes retrieved: a retrospective cohort study. Arch Gynecol Obstet 300 1791–1796. (10.1007/s00404-019-05373-9) 31705284

[bib13] Luo X, Li Y, Zheng H, et al. 2021 Thicker endometrium on hCG trigger day improves the live birth rate of fresh cleavage embryo transfer in GnRH-agonist regimen of women. Ann Transl Med 9 856. (10.21037/atm-21-1922) 34164490 PMC8184494

[bib14] Oron G, Hiersch L, Rona S, et al. 2018 Endometrial thickness of less than 7.5 mm is associated with obstetric complications in fresh IVF cycles: a retrospective cohort study. Reprod Biomed Online 37 341–348. (10.1016/j.rbmo.2018.05.013) 30146441

[bib15] Richter KS, Bugge KR, Bromer JG, et al. 2007 Relationship between endometrial thickness and embryo implantation, based on 1,294 cycles of in vitro fertilization with transfer of two blastocyst-stage embryos. Fertil Steril 87 53–59. (10.1016/j.fertnstert.2006.05.064) 17081537

[bib16] Shaodi Z, Qiuyuan L, Yisha Y, et al. 2020 The effect of endometrial thickness on pregnancy outcomes of frozen-thawed embryo transfer cycles which underwent hormone replacement therapy. PLoS One 15 e0239120. (10.1371/journal.pone.0239120) 32970718 PMC7513995

[bib17] Wu Y, Gao X, Lu X, et al. 2014 Endometrial thickness affects the outcome of in vitro fertilization and embryo transfer in normal responders after GnRH antagonist administration. Reprod Biol Endocrinol 12 96. (10.1186/1477-7827-12-96) 25296555 PMC4197319

[bib18] Xu DF, Liu PP, Fan L, et al. 2022 GnRH antagonist weakens endometrial stromal cells growth ability by decreasing c-kit receptor expression. Reprod Biol Endocrinol 20 29. (10.1186/s12958-021-00886-y) 35120552 PMC8815158

[bib19] Yang R, Guan Y, Perrot V, et al. 2021 Comparison of the long-acting GnRH agonist follicular protocol with the GnRH antagonist protocol in women undergoing in vitro fertilization: a systematic review and meta-analysis. Adv Ther 38 2027–2037. (10.1007/s12325-020-01612-7) 33651282 PMC8107074

[bib20] Yu J, Li B, Li H, et al. 2023 Comparison of uterine, endometrial and subendometrial blood flows in predicting pregnancy outcomes between fresh and frozen-thawed embryo transfer after GnRH antagonist protocol: a retrospective cohort study. J Obstet Gynaecol 43 2195937. (10.1080/01443615.2023.2195937) 37029723

[bib21] Zhang X, Chen CH, Confino E, et al. 2005 Increased endometrial thickness is associated with improved treatment outcome for selected patients undergoing in vitro fertilization-embryo transfer. Fertil Steril 83 336–340. (10.1016/j.fertnstert.2004.09.020) 15705371

[bib22] Zhu Q, Lin J, Gao H, et al. 2020 The Association between embryo quality, number of transferred embryos and live birth rate after vitrified cleavage-stage embryos and blastocyst transfer. Front Physiol 11 930. (10.3389/fphys.2020.00930) 32922305 PMC7456822

