# Supplementary material for: The association of endometrial thickness with live birth: a differential impact of agonist vs antagonist ovarian stimulation protocols
Source: Reprod Fertil. 2026 Sep 2;7(3):RAF250050. doi: 10.1530/RAF-25-0050 (PMC13548112; doi:10.1530/RAF-25-0050)
Supplement: Supplementary file 1 [file RAF-25-0050_supplementary_figures.pdf]

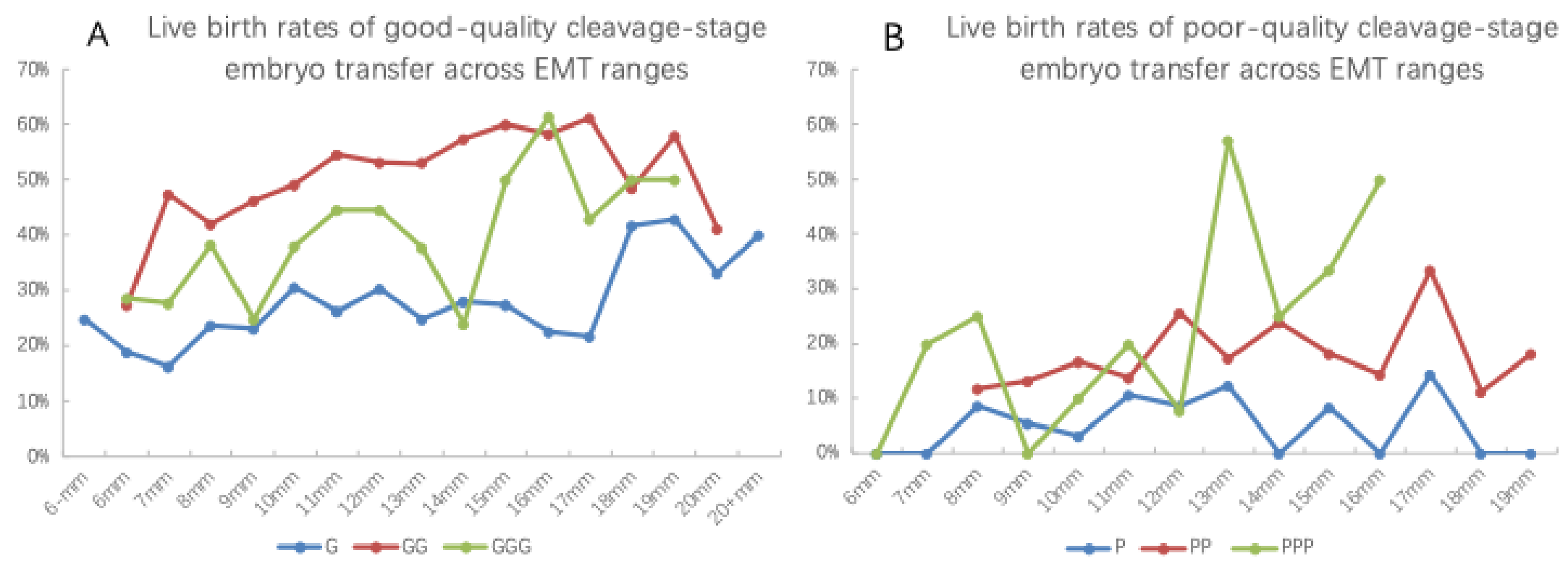

Figure S1. Live birth rate versus endometrial thickness with varying numbers of transferred embryos of same quality using agonist regimen. (A) Endometrial thickness on hCG Day showed a significant positive correlation with live birth rate in all transfer cycles involving good-quality cleavage-stage embryos (G: $\beta = 0.709$, $R^2 = 0.503$, $p = 0.001$; GG: $\beta = 0.525$, $R^2 = 0.276$, $p = 0.044$; GGG: $\beta = 0.673$, $R^2 = 0.453$, $p = 0.008$). (B) For poor-quality cleavage-stage embryos, a significant correlation was observed only with the transfer of three embryos (PPP: $\beta = 0.671$, $R^2 = 0.450$, $p = 0.024$), but not with single (P: $\beta = 0.016$, $R^2 < 0.001$, $p = 0.957$) or double (PP: $\beta = 0.216$, $R^2 = 0.047$, $p = 0.458$) embryos.

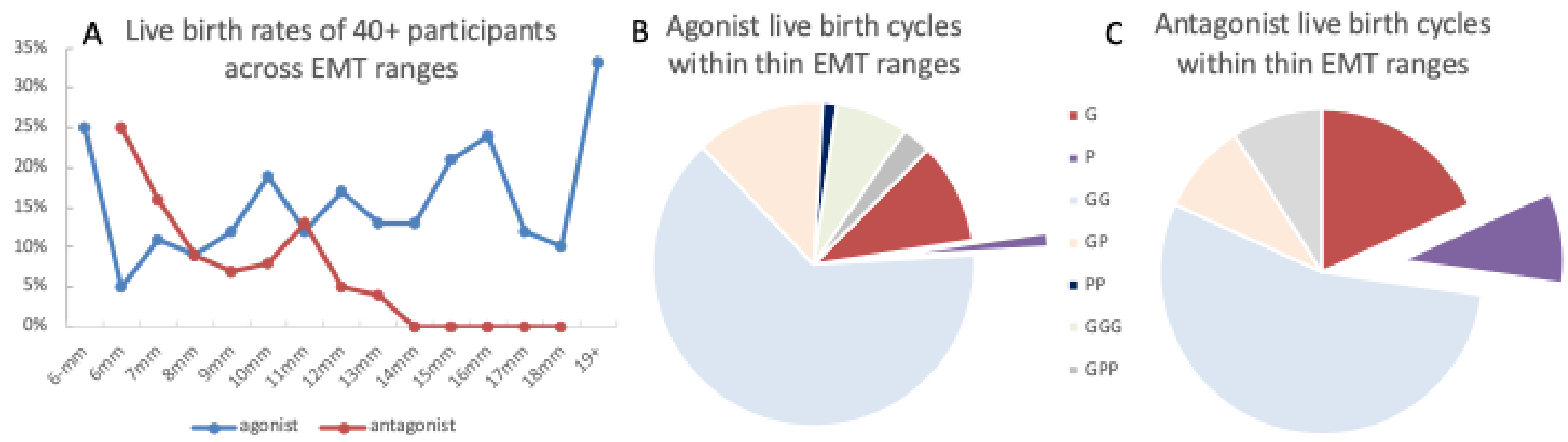

Figure S2. (A) Live birth rates among all women aged ≥40 years across different endometrial thickness (EMT) ranges, comparing agonist and antagonist groups.(B, C) Proportions of live birth cycles within the subgroup of women with thin endometrium (EMT ≤ 7 mm), in the (B) agonist or (C) antagonist group.
