# Supplementary material for: The association of endometrial thickness with live birth: a differential impact of agonist vs antagonist ovarian stimulation protocols
Source: Reprod Fertil. 2026 Sep 2;7(3):RAF250050. doi: 10.1530/RAF-25-0050 (PMC13548112; doi:10.1530/RAF-25-0050)
Supplement: Supplementary file 2 [file RAF-25-0050_supplementary_tables.pdf]

Table S1. Comparison of general characteristics between the live birth and non-live birth cycles within the agonist and antagonist groups. Values are presented as means ± standard deviations.

**Variables**	**Agonist live birth cycles**	**Agonist non-live birth cycles**	**P value**	**Antagonist live birth cycles**	**Antagonist non-live birth cycles**	**P value**
Female age (years)	31.46±4.01	33.24±4.73	< 0.001	30.91±4.23	32.95±5.60	< 0.001
Infertility year (years)	4.245±2.98	4.628±3.43	< 0.001	3.89±2.63	4.45±3.29	< 0.001
Baseline FSH (IU/L)	8.01±2.46	8.22±3.22	< 0.001	7.71±2.59	8.16±3.63	< 0.001
Baseline LH (IU/L)	5.08±3.97	4.80±3.14	0.006	6.31±4.27	6.11±4.97	0.275
Baseline AMH (ng/mL)	4.30±2.91	3.71±2.878	< 0.001	6.47±4.278	5.52±5.04	0.803
Gn dosage in COS (IU)	2221.89±789.69	2452.28±858.24	< 0.001	1663.46±661.60	1788.18±775.89	< 0.001
COS duration (day)	11.87±4.10	11.88±3.66	0.834	9.86±2.60	9.6±2.68	0.074
FSH on hCG Day (IU/L)	18.04±7.50	19.66±7.81	< 0.001	15.39±6.83	16.88±8.37	< 0.001
LH on hCG Day (IU/L)	1.83±1.30	1.84±1.79	0.001	3.5±3.482	3.58±3.99	0.273
P on hCG Day (ng/mL)	1.08±1.11	1.1±0.60	0.255	1.05±0.63	1.14±1.87	0.194
EMT on hCG Day (mm)	12.06±2.67	11.6±2.75	0.009	11.02±2.27	10.79±2.38	0.116
Oocytes retrieved	11.64±5.07	10.75±5.31	0.002	11.01±5.06	10.46±5.50	0.003
Embryo transferred	1.95±0.42	1.84±0.55	< 0.001	1.81±0.44	1.71±0.542	< 0.001

FSH = follicular stimulating hormone; LH = luteinizing hormone; AMH = anti-müllerian hormone; COS = controlled ovarian stimulation; hCG = human chorionic gonadotrophin; P = progesterone; EMT = endometrial thickness.

Table S2. Comparison of clinical pregnancy rate, live birth rate, multiple live birth rate of the subgroup between the agonist and antagonist groups.

	**Clinical pregnancy rate**			**Live birth rate**			**Multiple live birth rate**		
**Subgroup**	**Agonist**	**Antagonist**	**P value**	**Agonist**	**Antagonist**	**P value**	**Agonist**	**Antagonist**	**P value**
G	52.02%	44.46%	0.002	27.79%	22.84%	0.022	0.54%	0	0.077
P	35.96%	35.15%	0.835	17.73%	16.34%	0.650	0	0	NA

GG	68.43%	61.13%	< 0.001	52.07%	40.03%	< 0.001	18.27%	12.06%	< 0.001
GP	53.70%	41.59%	< 0.001	36.54%	25.08%	< 0.001	8.09%	3.80%	0.009
PP	36.65%	28.19%	0.050	21.12%	11.41%	0.007	4.98%	2.01%	0.112
GGG	53.33%	30.95%	0.006	38.27%	16.67%	0.006	13.58%	0	0.011
GGP	52.29%	32.14%	0.043	36.26%	10.71%	0.007	9.53%	3.57%	0.256
GPP	48.28%	46.15%	0.885	37.07%	30.77%	0.452	11.21%	15.38%	0.464
PPP	33.78%	38.46%	0.486	24.32%	7.69%	0.166	5.40%	0	0.517

Table S3. Variables not included in the binary logistical regression model

Agonist Group					Antagonist Group				
Variable	β	OR	95% CI	P	Variable	β	OR	95% CI	P
Baseline FSH	-0.001	0.999	0.982-1.016	0.946	Baseline FSH	-0.014	0.986	0.95-1.022	0.436
Baseline AMH	0.006	1.006	0.985-1.027	0.591	Baseline AMH	0.003	1.003	0.978-1.028	0.83
Gn dosage in COS	0.005	1.005	0.987-1.018	0.938	Gn dosage in COS	0.001	1.001	0.980-1.021	0.097
COS duration	0.016	1.016	0.99-1.042	0.217	COS duration	0.096	1.100	0.993-1.017	0.062
FSH on hCG Day	0.002	1.002	0.992-1.012	0.097	FSH on hCG Day	0.002	1.002	0.983-1.019	0.963
LH on hCG Day	-0.006	0.994	0.959-1.029	0.721	LH on hCG Day	0.012	1.012	0.981-1.043	0.453
P on hCG Day	-0.011	0.989	0.923-1.055	0.745	P on hCG Day	0.002	1.002	0.994-1.01	0.588
Oocytes retrieved	0.005	1.005	0.993-1.017	0.372	Oocytes retrieved	-0.013	0.987	0.966-1.008	0.257

β = standard regression coefficient; OR = odds ratio; CI = confidence interval.

Table S4. The P value of the comparison of clinical pregnancy rates, live birth rates, and multiple birth rates between different subgroups from Dunn's T3 in agonist group. The blastocyst transfer cycles GGG, GGP, GPP were not included due to their small sample size.

Cleavage-Stage Embryo				Blastocyst			
	CPR	LBR	MBR		CPR	LBR	MBR
P vs PP	0	0.01	0	P vs PP	0.992	0.257	0.002
P vs PPP	0.471	0.485	0.933	P vs PPP	0.515	0.126	0.980
PP vs PPP	1	1	1	PP vs PPP	0.684	0.237	1
G vs GG	0	0	0	G vs GG	0.863	0.023	0.025
G vs GGG	0.465	0.01	0	G vs GP	0.865	0.999	0.021
GG vs GGG	0	0	0.289				
G vs GP	0.024	0	0				
G vs GPP	1	0.574	0.011				
GP vs GPP	1	1	1				

CPR = clinical pregnancy rate; LBR = live birth rate; MBR = multiple birth rate.

Table S5. The P value of the comparison of clinical pregnancy rates, live birth rates, and multiple birth rates between different subgroups from Dunn's T3 in the antagonist group.

Cleavage-Stage Embryo				Blastocyst			
	CPR	LBR	MBR		CPR	LBR	MBR
P vs PP	0.937	1	0.996	P vs PP	0.997	1	0.032
P vs PPP	1	1	1	G vs GG	0.000	0.000	1
PP vs PPP	1	1	0.996	G vs GP	1	1	0.771
G vs GG	0	0	0				
G vs GGG	1	1	1				
GG vs GGG	0.003	0.01	0				
G vs GP	1	1	0.051				
G vs GPP	1	1	0.971				
GP vs GPP	1	1	0.998				

CPR = clinical pregnancy rate; LBR = live birth rate; MBR = multiple birth rate.

Table S6. Correlation analysis between EMT and live birth on the ROC curve in different subgroups of embryo transfer cycles using GnRH-antagonist regimen.

Cleavage stage embryo					Blastocyst				
Subgroup	AUC	P value	95% CI	Cutoff (mm)	Subgroup	AUC	P value	95% CI	Cutoff(mm)
G	0.535	0.309	0.469-0.601	11.65	G	0.398	0.052	0.302-0.495	7.1
P	0.307	0.200	0.045-0.568	7.15	P	0.492	0.890	0.380-0.604	9.55
GG	0.537	0.025	0.505-0.568	9.05	GP	0.67	0.298	0.381-0.959	11
GP	0.584	0.030	0.504-0.664	11.95	PP	0.423	0.591	0.219-0.627	6
PP	0.441	0.507	0.280-0.603	9.25					
GGG	0.41	0.458	0.183-0.637	13.6					
GGP	0.713	0.235	0.536-0.891	9.9					
GPP	0.819	0.076	0.583-1	9.5					
PPP	0.5	1.000	0.203-0.797	9.5					

AUC = area under the curve; CI = confidence interval.
